# Dendritic Cells Expressing Triggering Receptor Expressed on Myeloid Cells-1 Correlate with Plaque Stability in Symptomatic and Asymptomatic Patients with Carotid Stenosis

**DOI:** 10.1371/journal.pone.0154802

**Published:** 2016-05-05

**Authors:** Vikrant Rai, Velidi H. Rao, Zhifei Shao, Devendra K. Agrawal

**Affiliations:** Department of Clinical and Translational Science, Creighton University School of Medicine, Omaha, Nebraska 68178, United States of America; National Jewish Health, UNITED STATES

## Abstract

Atherosclerosis is a chronic inflammatory disease with atherosclerotic plaques containing inflammatory cells, including T-lymphocytes, dendritic cells (DCs) and macrophages that are responsible for progression and destabilization of atherosclerotic plaques. Stressed cells undergoing necrosis release molecules that act as endogenous danger signals to alert and activate innate immune cells. In atherosclerotic tissue the number of DCs increases with the progression of the lesion and produce several inflammatory cytokines and growth factors. Triggering receptor expressed on myeloid cells (TREM)-1 plays a crucial role in inflammation. However, relationship of DCs and the role of TREM-1 with the stability of atherosclerotic plaques have not been examined. In this study, we investigated the heterogeneity of the plaque DCs, myeloid (mDC1 and mDC2) and plasmacytoid (pDCs), and examined the expression of TREM-1 and their co-localization with DCs in the plaques from symptomatic (S) and asymptomatic (AS) patients with carotid stenosis. We found increased expression of HLA-DR, fascin, and TREM-1 and decreased expression of TREM-2 and α-smooth muscle actin in S compared to AS atherosclerotic carotid plaques. Both TREM-1 and fascin were co-localized suggesting increased expression of TREM-1 in plaque DCs of S compared to AS patients. These data were supported by increased mRNA transcripts of TREM-1 and decreased mRNA transcripts of TREM-2 in carotid plaques of S compared to AS patients. There was higher density of both CD1c+ mDC_1_ and CD141+ mDC_2_ in the carotid plaques from AS compared to S patients, where as the density of CD303+ pDCs were higher in the carotid plaques of S compared to AS patients. These findings suggest a potential role of pDCs and TREM-1 in atherosclerotic plaque vulnerability. Thus, newer therapies could be developed to selectively block TREM-1 for stabilizing atherosclerotic plaques.

## Introduction

Cardiovascular disease is currently the principal cause of death in the western world [1–2]. Clinical symptoms in occlusive arterial diseases, including myocardial infarction, stroke, and lower limb ischemia, are all related to a common denominator, atherosclerosis [2]. Atherosclerosis is a chronic cardiovascular inflammatory disease initiated by an infiltration of low-density lipoprotein cholesterol (LDL) into the intimal layer of the artery. The presence of LDL within the arterial wall leads to endothelial dysfunction and subsequent recruitment of leukocytes ultimately resulting in the formation of atheroma or plaque that can grow in size and cause occlusion of the arterial lumen [3–4]. Integral to the progression of atherosclerotic plaque formation are the innate and adaptive immune responses and misdirected activation of immune system mediating the chronic inflammatory process in the arterial wall [2, 4–6]. The recruited leukocytes include monocytes/macrophages, dendritic cells (DCs) and activated T cells. Recruitment of higher number of monocytes and T lymphocytes potentiates the inflammatory process. Compared to stable plaque, vulnerable plaque has enhanced inflammation and it plays a crucial role in plaque destabilization [6–8]. Libby et al. [9] reported that thrombogenicity of the plaque and integrity of the fibrous cap of the plaque is regulated by inflammation and it provides a correlation between inflammation and thrombotic complications.

Although macrophages are the prominent phagocytic cells in the intima, the presence of DCs has also been highlighted [3–4]. Indeed, there is heterogeneity within the phagocytic cell population in the plaque that exhibit phenotypic and functional traits of DCs, including the expression of DC markers and the capacity for antigen presentation and stimulation of T cell activation [3]. Vulnerable plaques have significantly higher number of total and mature DCs in the shoulder region and slightly increased number of mature DCs in the fibrous cap region [8]. DCs are crucial for inducing but also dampening immune responses, therefore, it is crucial to determine the heterogeneity of DCs to further elucidate the implication of DC subset distribution to the pathophysiology of atherosclerosis and plaque instability. In vivo, several different subsets of DCs can be identified based on cell-surface expression markers, location and function [4, 10–11]. We have previously shown an increase in apoptosis and inflammation in human atherosclerotic plaques from symptomatic relative to asymptomatic patients with carotid stenosis [6, 12]. Recently discovered triggering receptor expressed on myeloid cells-1 (TREM-1) is involved in inflammatory and immune response, and expressed on neutrophils, monocytes and bone marrow-derived DCs [13–15]. Association of TREM-1 with hypoxia and inflammation and their presence in atherosclerotic area has been reported. This association results in secretion of pro-inflammatory cytokines and reactive oxygen species [13, 16–19]. Hence, TREM-1 might play a crucial role in plaque vulnerability.

It is evident that inflammation, lipid accumulation, proteolysis, thrombosis, angiogenesis and apoptosis play an important role in plaque vulnerability and hence, many serum markers for these molecular processes have been examined. These markers include C-reactive protein [20], myeloperoxidase [21], serum myeloid-A [22], matrix metalloproteinases (MMPs) [23], interleukin (IL)-18, IL-4, IL-6, IL-8, IL-12, transforming growth factor -β1, interferon (IFN)-γ, tumor necrosis factor (TNF)-α, vascular endothelial growth factor and regulated on activation, normal T cell expressed and secreted (RANTES) [24–26]. These studies have been performed in both coronary and carotid artery diseases, but the results in carotid artery atherosclerosis are inconclusive. Also, the assessment of transpiring nature of asymptomatic to symptomatic plaque is difficult; hence there is a need of novel non-invasive biochemical marker for carotid plaque instability. In this study, we examined the plasmacytoid DC (pDC) and myeloid DCs (mDC1 and mDC2) subsets in carotid plaques and compared these two subsets in the plaques of symptomatic and asymptomatic patients with carotid artery disease. We also examined the expression pattern of TREM-1, human leukocyte antigen (HLA)-DR, and fascin by immunofluorescence, with alpha-smooth muscle actin (α-SMA) as a selective marker to identify vascular smooth muscle cells (VSMCs) in plaque DCs and compared between the plaques from S and AS patients with carotid artery stenosis.

## Materials and Methods

### Carotid endarterectomy specimen

The Institutional Review Board of Creighton University approved the research protocol as an exempt status since all tissue samples were collected in an anonymous manner, as has been used in recent studies [27, 28]. No specimen of the carotid endarterectomy biopsy tissue was marked or identified by patient or medical record number, pathology accession number, social security number, or any other identifiable information. None of the research investigators could identify the patients from whom the carotid endarterectomy tissues were obtained. The Institutional Review Board of Creighton University waived the need for consent. The operating surgeon collected surgical specimens of human atherosclerotic plaques while operating on the patients undergoing carotid endarterectomy procedures. The operating surgeon categorized the carotid endarterectomy specimens as either symptomatic (S) or asymptomatic (AS) according to patients’ history, symptoms, and clinical examination. The research investigators were provided the specimens without any identifiable information of the patient, but the information of S or AS plaques, age, sex and ethnicity was provided.

In this study, we utilized surgical specimens of human atherosclerotic plaques (plaques from 14 asymptomatic patients, 64.0 ± 6.6 years, and plaques from 14 symptomatic patients, 74.0 ± 5.3 years; both male and female) were obtained from patients undergoing carotid endarterectomy procedures for symptomatic disease (including hemispheric transient ischemic attack, amaurosis fugax or stroke) and asymptomatic high-grade severe stenosis. Carotid endarterectomy tissues were collected in the University of Wisconsin (UW) solution and transported to the lab and maintained at 4°C. This solution maintains the functional and morphological integrity of the vascular specimen for at least 24 hours [29].

### Preparation and staining of specimen

Carotid specimens were fixed in 4% formalin and each specimen was transversely sectioned at 2mm and embedded in paraffin. Thin sections (5μm) were cut using a microtome and stained with hematoxylin and eosin (H&E) following manufacturer’s standard protocol (Newcomer/supply).

### Immunofluorescence study

We performed deparaffinization, rehydration and antigen retrieval prior to immunostaining. The thin sections on the slides were given the PBS washings and then incubated for one hour in blocking solution/permeabilizing solution containing 5% (v/v) of appropriate serum with 0.25% Triton X-100 and 0.1% bovine serum albumin. The slides were subsequently incubated with primary antibody including mouse anti-human HLA-DR (SantaCruz; sc-73366) at 1:50 dilution, mouse anti-fascin (Dako; M3567) at 1:100 dilution, mouse anti α-SMA at 1:200 dilution (abcam; ab7817), goat anti-TREM 1 (SantaCruz Biotech; sc-19309) and rabbit anti-TREM 2 (SantaCruz Biotech; sc-48764) at 1:50 dilution and allowed to bind at room temperature for 2 hours. The sections were washed with PBS and incubated with Alexa Fluor 594 (red) and Alexa Fluor 488 (green)-conjugated secondary antibodies (Invitrogen, Grand Island, NY, USA) for 1 hour (1:1000 dilution) at room temperature. The slides were washed with PBS and counterstained with DAPI (4, 6-diamidino-2-phenylindole) to stain nuclei. Immunofluorescence microscopy was done with an Olympus inverted fluorescent microscope (Olympus BX51). Negative controls were run by using the isotype antibody. Three images of each section were taken and used for the calculation of fluorescence intensity of TREM-1, TREM-2, HLA-DR, fascin, and α-SMA by Image-J software (NIH) in each experimental group. The average of the three values was calculated for the analyzing the mean fluorescence intensity of the sample in each group. Similarly, for counting the number of positive cells for TREM-1, TREM-2, fascin, HLA-DR and α-SMA, three images from each sample were randomly taken and the total number of cells were counted in each image. The average of the cell numbers from these three images was calculated and considered as the number of cells per field of view. A total of five samples in each group were used to calculate the number of positive cells.

### Isolation and Characterization of the Plaque DCs

The isolation of mDC_1_ (CD11+CD1c+MHCII+CD123-), mDC_2_ (CD11+CD1c-CD141+MHC+CD123-) and pDCs (CD123+CD303+CD11-CD1c-) was performed by established methods in our laboratory. Briefly, carotid plaques were finely chopped into small fragments and were digested with collagenase D in RPMI-1640 containing DNase at 37°C for 4 hours. The digested tissue was dissociated to obtain the plaque cell suspension. The entire plaque cells were filtered with a 100μm cell strainer to remove large tissue debris. The small crystallized or calcified debris with higher density was removed by gradient centrifugation. The cell suspension was then filtered with 40μm cell strainer. The filtered cells were washed with PBS and counted before immunostaining. Cells were re-suspended in PBS supplemented with 4% FBS and incubated with following anti-human antibodies for 30 minutes on ice: CD1c for mDC_1_, CD141 for mDC_2_ and CD303 for pDCs. The pDCs in symptomatic carotid plaques were further confirmed as CD123+ and sorted cells were checked for immunoreactivity for CD1c.

### RNA isolation, cDNA synthesis, and real-time PCR

Total mRNA isolation was done by using TRI reagent (Trizol reagent, Sigma, St Louis, MO, USA) from tissues according to the manufacturer’s instructions. The Nanodrop (Thermo Scientific, Rockford, IL, USA) was used for RNA quantitation. Further, the cDNA was synthesized using Improm II reverse transcription kit (Promega, Madison, WI, USA) following the manufacturer’s instructions. Real-time PCR (RT-PCR) was performed in triplicate using SYBR Green Master Mix and a Real-time PCR system (CFX96, BioRad Laboratories, and Hercules, CA, USA). The primers for different genes were obtained from Integrated DNA Technologies. (Coralville, IA, USA). The PCR cycling conditions were 5 min at 95°C for initial denaturation, 40 cycles of 30s at 95°C, 30s at 55–60°C (according to the primer annealing temperatures) and 30s at 72°C followed by melting curve analysis. Fold expression of mRNA transcripts relative to controls was determined after normalizing to glyceraldehyde-3-phosphate dehydrogenase (GAPDH). The oligonucleotide primer sequences for TREM-1 and TREM-2 and GAPDH were as follows. TREM-1: AGT TAC AGC CCA AAA CAT GC (Forward primer 5’-3’) and CAG CCC CCA CAA GAG AAT TA (Reverse primer 5’-3’); TREM-2: ACA GAA GCC AGG GAC ACA TC (Forward primer 5’-3’) and CCT CCC ATC ATC TTC CTT CA (Reverse primer 5’-3’); and GAPDH: GGT GAA GGT CGG AGT CAA CGG ATT TGG TCG (Forward primer 5’-3’) and GGA TCT CGC TCC TGG AAG ATG GTG ATG GG (Reverse primer 5’-3’).

### Statistical analysis

All the data are presented as mean ± SD. Differences between AS and S group was analyzed by Student’s t-test. A value of p<0.05 was considered statistically significant. *p <0.05, **p <0.01,***p <0.001 and ****p <0.0001.

## Results

### Neo-intima and plaque formation in symptomatic patients

**Fig 1:**
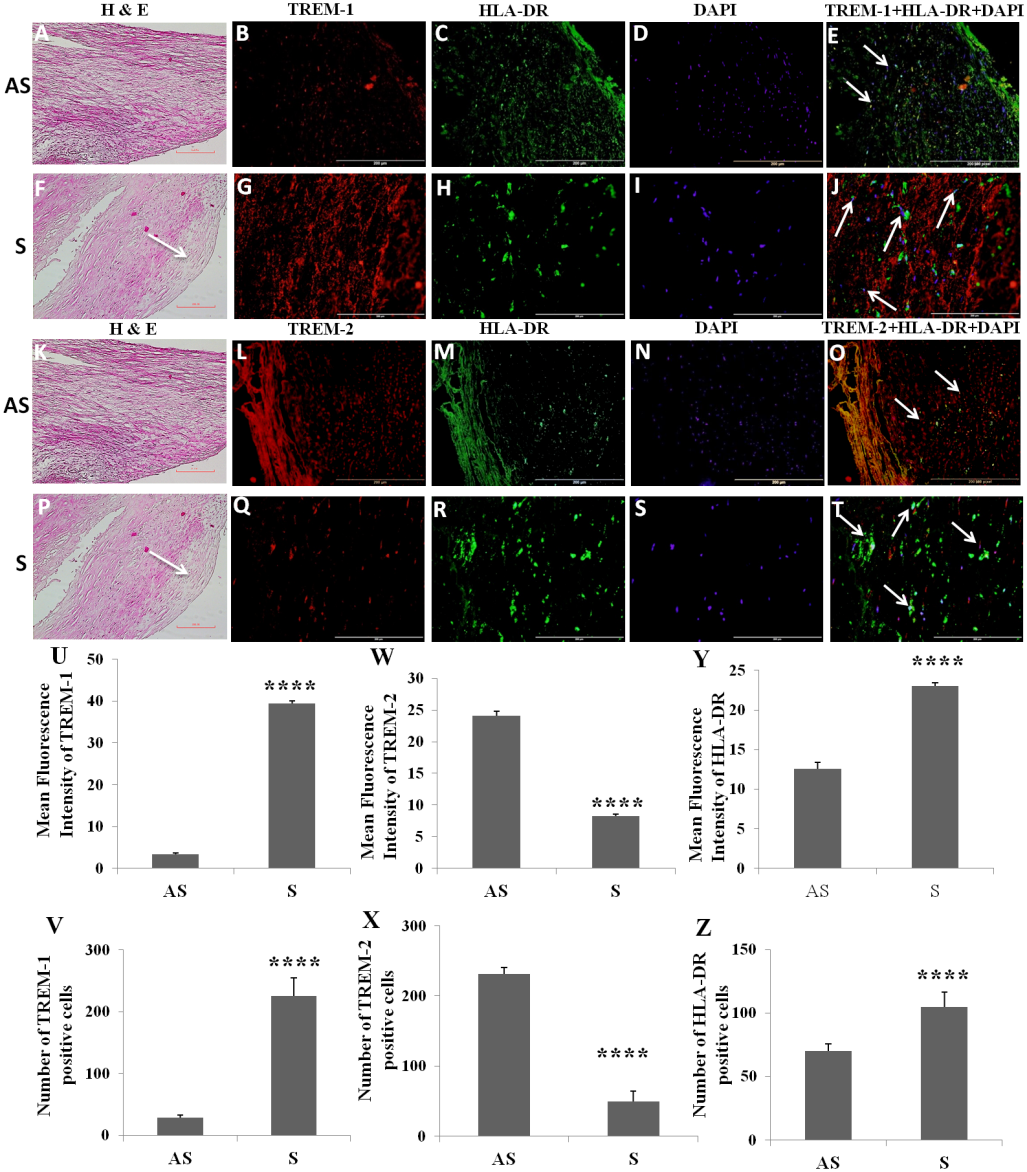
Immunofluorescence staining for TREM-1, TREM-2, and HLA-DR and co-localization of TREMs with HLA-DR+ cells in carotid plaques. H&E staining shows the histology of carotid plaques in AS (panels A, K) and S (panels F, P) patients. Immunofluorescence staining for TREM-1 (panels B, G), TREM-2 (panels L, Q), HLA-DR (panels C, H, M, R), DAPI (panels D, I, N, S) was performed and merged for examining co-localization of TREM-1 and HLA-DR (panels E, J) and for TREM-2 and HLA-DR (panels O, T). Arrows shows the co-localization of TREM-1 and TREM-2 with HLA-DR. These are the representative images of 3 randomly selected samples from independent patients in each experimental group. Mean fluorescence intensity of TREM-1 (panel U), TREM-2 (panel W) and HLA-DR (panel Y) and number of positive cells for TREM-1 (panel V), TREM-2 (panel X) and HLA-DR (panel Z). Data are shown as mean ± SD (N = 3); *p<0.05, **p<0.01, ***p<0.001 and ****p<0.0001.

H&E staining of the carotid plaque tissue from S and AS patient showed neo-intima and plaque formation in symptomatic carotid plaques (Fig 1F and 1P). Decreased cellularity was observed in the S compared to AS carotid plaques (Fig 1).

### Increased expression of TREM-1 and HLA-DR and decreased expression of TREM-2 in carotid plaques of symptomatic patients

HLA-DR+ cells were localized to the sub-endothelial area in the intima layer of both symptomatic and asymptomatic atherosclerotic carotid plaques. However, in addition to the sub-endothelial layer location, HLA-DR+ cells were widely distributed in the atherosclerotic plaque from symptomatic and asymptomatic patients with carotid stenosis (Fig 1). These HLA-DR+ cells were significantly greater in the areas of thin fibrous cap and shoulder of the S compared to AS plaques (Fig 1C, 1H, 1M and 1R). There was significantly increased expression of TREM-1 (Fig 1B, 1G, 1U and 1V) and HLA-DR (Fig 1C, 1H, 1M, 1R, 1Y and 1Z), and significantly decreased expression of TREM-2 (Fig 1L, 1Q, 1W and 1X) in the S compared to AS plaques. TREM-1 and TREM-2 were found to be co-localized with HLA-DR+ cells (Fig 1E, 1J, 1O and 1T).

### Increased expression of Fascin, decreased expression of TREM-2, and co-localization of TREMs in Fascin+ cells in carotid plaque of symptomatic patients

**Fig 2:**
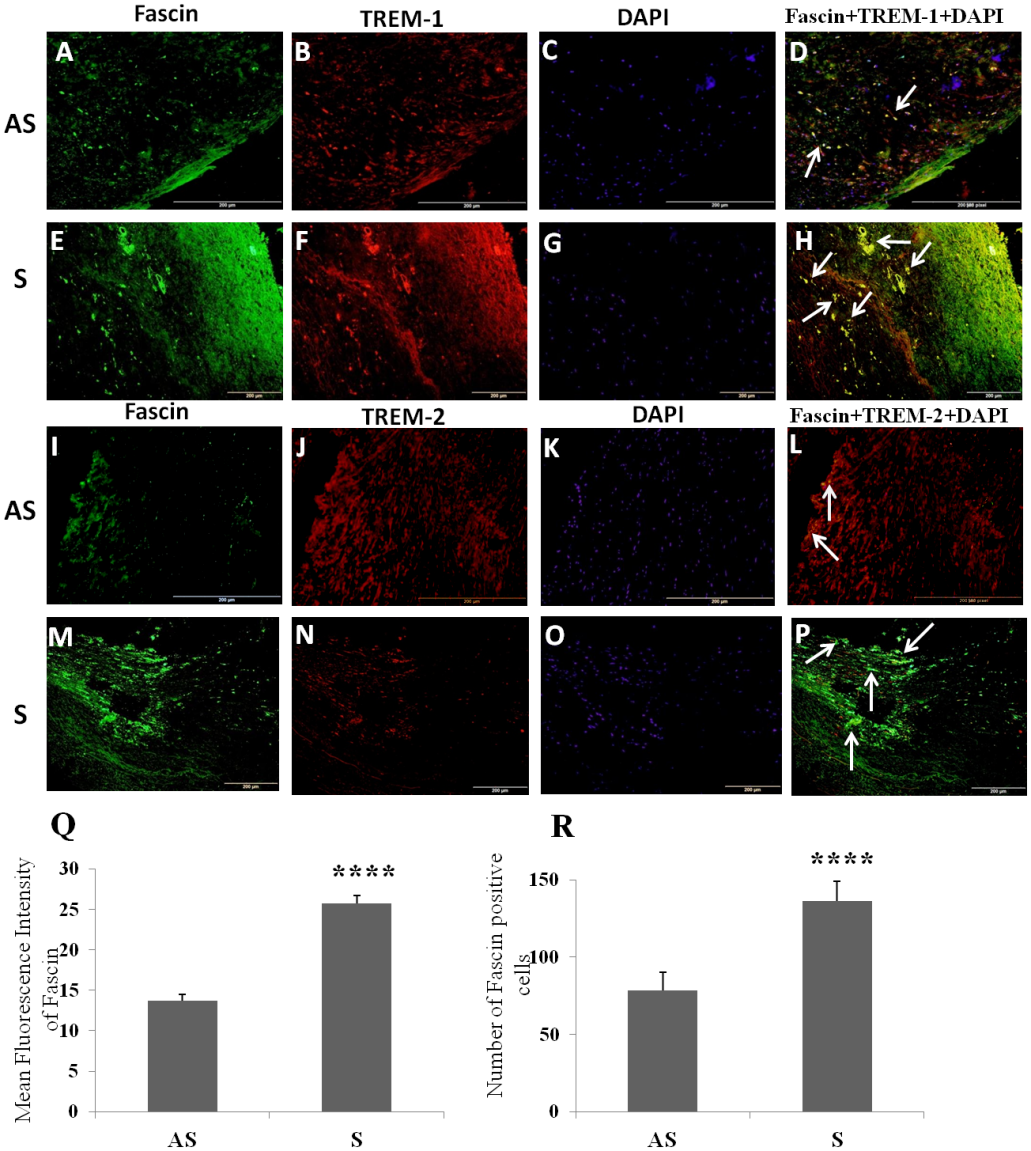
Immunofluorescence staining for Fascin and TREM-2, and co-localization of TREMs with Fascin+ cells in carotid plaques. Immunofluorescence staining for TREM-1 (panels B, F), TREM-2 (panels J, N), fascin (panels A, E, I, M), DAPI (panels C, G, K, O) was performed and merged for examining the co-localization of TREM-1 and fascin (panels D, H) and for TREM-2 and fascin (panels L, P). Arrows shows the co-localization of TREM-1 and TREM-2 with fascin. These are the representative images of 3 randomly selected samples in each experimental group. Mean fluorescence intensity for fascin (panel Q) and number of positive cells for fascin (panel R). Data are shown as mean ± SD (N = 3); *p<0.05, **p<0.01, ***p<0.001 and ****p<0.0001.

Fascin+ cells were mainly localized to sub-endothelial area of the intima layer of both AS and S atherosclerotic carotid plaques. However, in addition to the subendothelial layer location, fascin+ cells were widely distributed in the atherosclerotic plaques from S and AS patients with carotid stenosis (Fig 2). These fascin+ cells were significantly greater in the areas of thin fibrous cap and shoulder region of S compared to AS plaques (Fig 2A, 2E, 2I and 2M). There was significantly increased expression of TREM-1 (Fig 2B and 2F), fascin (Fig 2A, 2E, 2I, 2M, 2Q and 2R) and significantly decreased expression of TREM-2 (Fig 2J and 2N) in the S compared to AS plaques. TREM-1 and TREM-2 were found to be co-localized with fascin+ cells (Fig 2D, 2H, 2L and 2P).

### Decreased expression of α-SMA and co-localization with TREMs in carotid plaques of symptomatic patients

**Fig 3:**
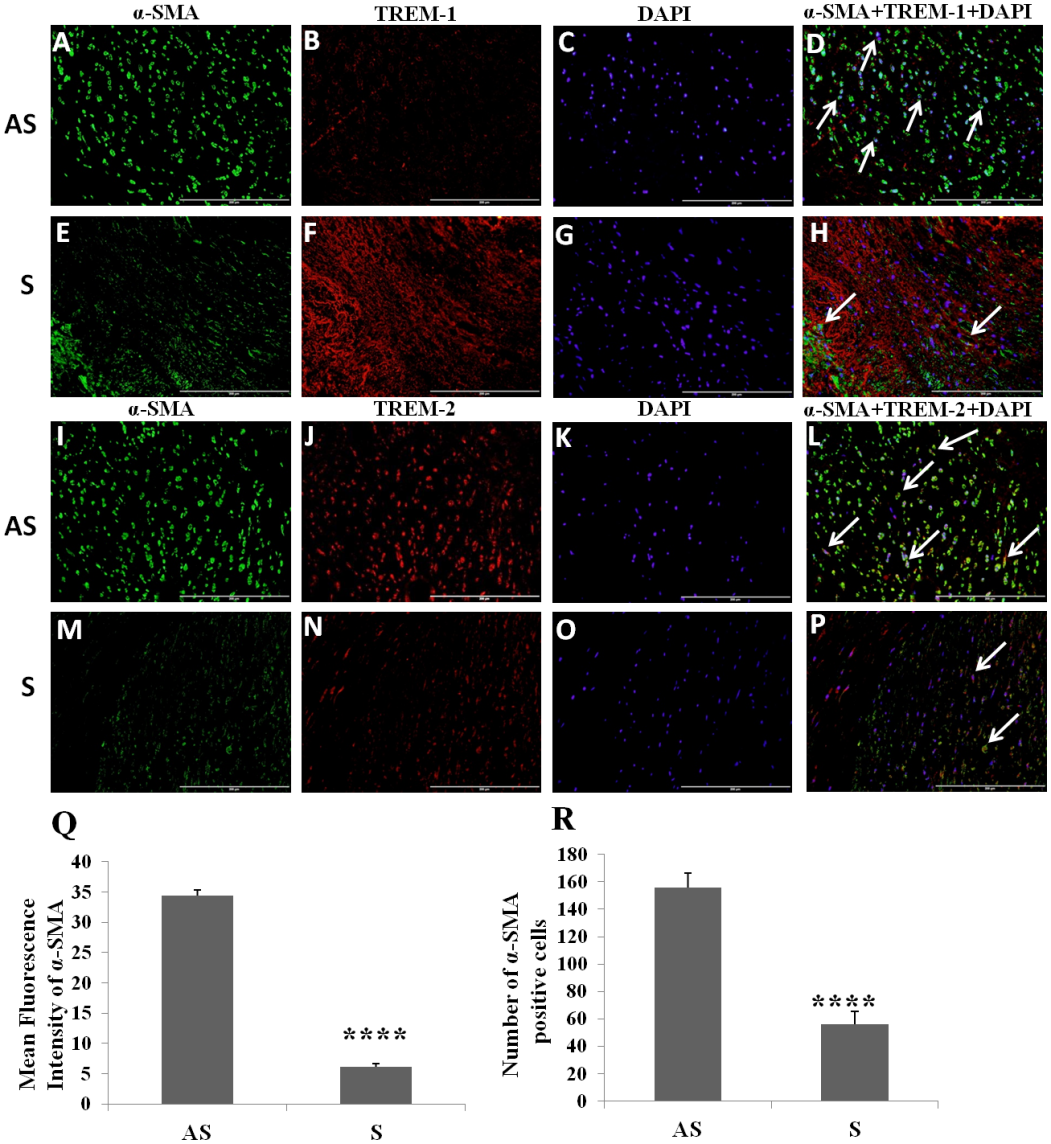
Immunofluorescence staining for α-SMA+ cells and co-localization of α-SMA+ cells with TREM-1 and TREM-2 in carotid plaques. Immunofluorescence staining for TREM-1 (panels B, F), TREM-2 (panels J, N), α-SMA (panels A, E, I, M), DAPI (panels C, G, K, O) was performed and merged for examining the co-localization of TREM-1 and α-SMA (panels D, H) and for TREM-2 and α-SMA (panels L, P). Arrows show the co-localization of TREM-1 and TREM-2 with α-SMA. These are the representative images of 3 randomly selected samples in each experimental group. Mean fluorescence intensity for α-SMA (panel Q) and number of positive cells for fascin (panel R). Data are shown as mean ± SD (N = 3); *p<0.05, **p<0.01, ***p<0.001 and ****p<0.0001.

Vascular smooth muscle cells were widely distributed in both symptomatic and asymptomatic atherosclerotic plaques (Fig 3). However, the VSMCs density was significantly lower in S (Fig 3E and 3M) compared to AS (Fig 3A and 3I). There was significantly increased expression of TREM-1 (Fig 3B and 3F), and significantly decreased expression of TREM-2 (Fig 3J and 3N) and α-SMA (Fig 3A, 3E, 3I, 3M, 3Q and 3R) in the S compared to AS plaques. TREM-1 and TREM-2 were found to be co-localized with α-SMA+ cells (Fig 3D, 3H, 3L and 3P).

### Increased mRNA expression of TREM-1 in carotid plaques of symptomatic patients

**Fig 4:**
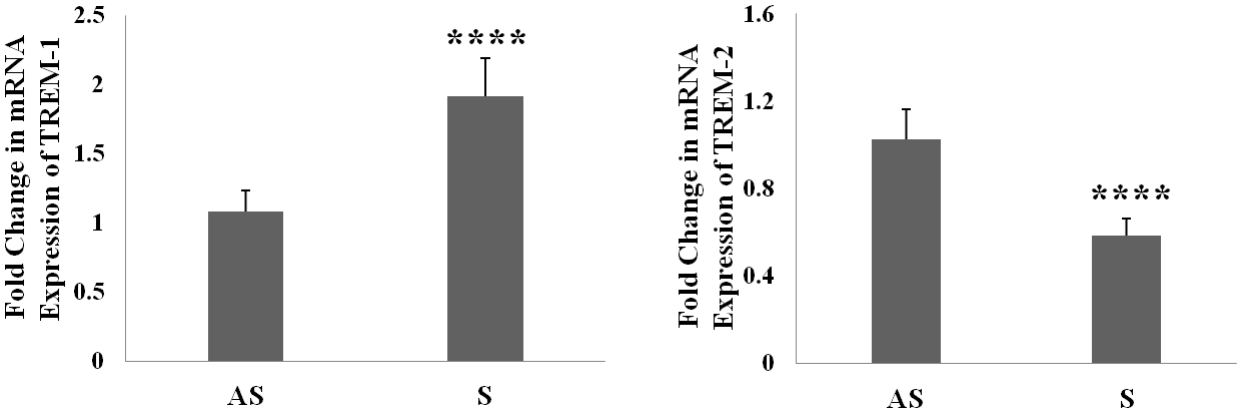
TREM-1 and TREM-2 mRNA expression analysis by RT-PCR. The cDNA prepared from mRNA was subjected to RT-PCR with gene specific primers (A: TREM-1; B: TREM-2) and the fold-change expression relative to GAPDH as housekeeping gene are shown. Results are expressed as fold-change in S compared to AS. Data are shown as mean ± SD (N = 6); *p<0.05, **p<0.01, ***p<0.001 and ****p<0.0001.

In tissue extracts of carotid plaques of symptomatic and asymptomatic patients, the mRNA expression of TREM-1 was higher (Fig 4A) whereas mRNA expression of TREM-2 was lower (Fig 4B) in S compared to AS. These results confirm our immunofluorescence results on increased expression of TREM-1 and decreased expression of TREM-2 in S compared to AS.

### Increased plasmacytoid DCs in symptomatic carotid plaques

**Fig 5:**
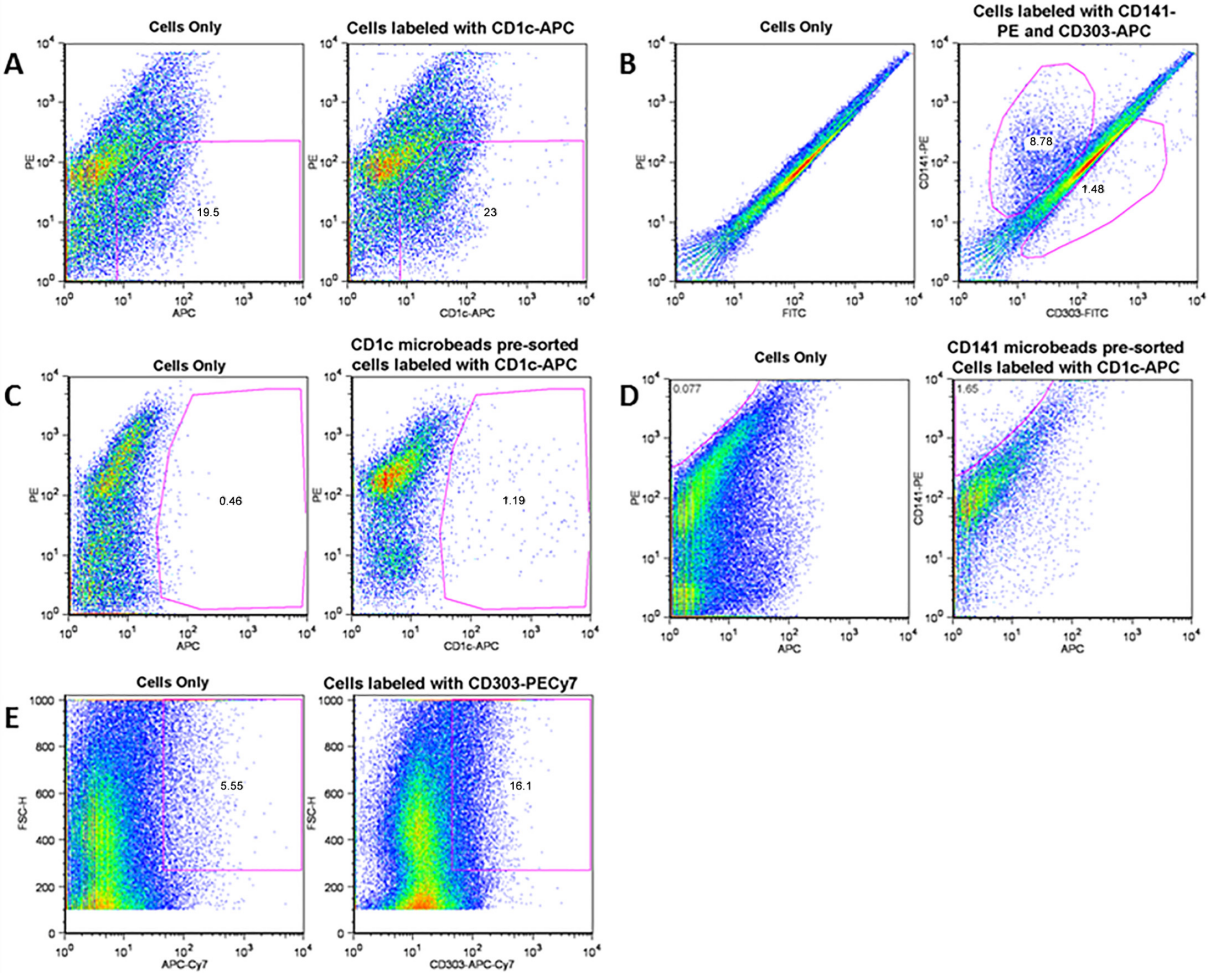
Dynamics of myeloid and plasmacytoid dendritic cells in S and AS carotid plaques. Cell characterization was performed using anti-human antibodies for mDC_1_ (CD1c+), mCD_2_ (CD141+) and pDC (CD303+). (A) Cells only are without antibody (background) and cells labeled with CD1_C_ antibody for mDC_1_. After subtracting background, mDC_1_ cells were 3.5%; (B) CD141+ cells (mDC_2_) were 8.78% and CD303+ cells (pDCs) were 1.41%; and (C) Cells only are without antibody (background) and cells labeled with CD1_C_ antibody for mDC_1_. After subtracting background, (D) mDC_1_ cells were 0.73% and CD141+ mDC_2_ were 1.58%, and (E) CD303+ cells (pDCs) were 10.7%. The data represent 4 plaques from independent patients in each experimental group.

In asymptomatic carotid plaques, the majority of the DCs had a phenotype of mDC_1_ and mDC_2_ with a few pDCs (Fig 5A and 5B; Table 1), however in symptomatic carotid plaques the majority of the DCs had a phenotype of pDCs and a very few mDC_1_ and mDC_2_ (Fig 5C, 5D and 5E; Table 1). The pDCs in S carotid plaques were further confirmed as CD123+ and sorted cells were checked for any immunoreactivity to CD1_C_. The CD303+CD123+ cells from this population of DCs primarily consisted of pDCs.

**Table 1:** Dynamics of dendritic cell phenotype in atherosclerotic carotid plaques of asymptomatic and symptomatic carotid stenosis patient.

Plaque DC Phenotype	Asymptomatic Carotid Plaque	Symptomatic Carotid Plaque
	Percent and (absolute number of total cells)	Percent and (absolute number of total cells)
CD1c+ mDC_1_	3.9–4.7% (390,000–470,000)	0.5–0.8% (50,000–80,000)
CD141+ mDC_2_	7.6–9.2% (760,000–920,000)	1.3–1.8% (130,000–180,000)
CD303+ pDC	0.2–1.6% (20,000–160,000)	9.6–15.3% (960,000–1,530,000)

## Discussion

Atherosclerosis, a chronic inflammatory cardiovascular process results in atheroma or plaque formation and recruitment of inflammatory cells such as macrophage, neutrophils and DCs [3–8]. Recent evidence in human indicates that the number of DCs increases with the progression of the atherosclerotic lesion and accumulation of mature DCs is observed in rupture-prone vulnerable plaques [3–4]. Among all type of APCs identified in the intimal layer of arterial wall, DCs are the most potent APCs due to their unique ability to induce primary immune response [30, 31]. APCs within the plaque are involved in the formation of foam cells and promote T helper-1 (TH_1_)-driven immune response [32]. DCs are emerging as a prime candidate in the pathophysiology of atherosclerosis due to their potent ability to present antigen and likely role in directing the innate or adaptive immune response against endogenous and exogenous antigen including ox-LDL, oxidized epitope on apoptotic cells, heat shock proteins and degradative products of extracellular matrix in atherosclerotic plaque and consequent plaque progression [4, 32]. In atherosclerotic artery DCs migrate (migrating myeloid DCs, HLA-DR+) to the peripheral region and there in an enhanced migration of the matured DCs (fascin+) in vulnerable plaque [8]. In this study, we found increased expression of TREM-1, HLA-DR and fascin and co-localization of HLA-DR and fascin with TREM-1 in S compared to AS patients with carotid stenosis, suggesting TREM-1-ionduced activation of DCs in unstable plaques.

The immune and immune competent cells in the intima form vascular-associated lymphoid tissue, which is responsible for testing the danger signals in the intima and DCs are the main element of vascular-associated lymphoid tissue. Studies have reported an alteration between the interactions of DCs and other infiltrated cells in the atherosclerosis pre-disposed area of vessel, indicating the possibility of crucial role of DCs in atherosclerosis. The vascular DCs are positive for HLA-DR and CD1a expression and are mainly present in the superficial layer of the intima [30]. HLA-DR+ cells are widely distributed in the intima of arterial wall and their density is more in the middle layer of the intima than the superficial and the deep layer. HLA-DR is a molecule of MHC II and is involved in the presentation of protein antigen to CD4+ lymphocytes, resulting in the initiation of immune response [30]. In this study, we found that the HLA-DR+ cells were mainly located in the sub-endothelial areas in the intima layer of both S and AS atherosclerotic carotid plaques, however, the fascin expression was expressed discretely in the inflammatory cell population but more near the intimal layer of plaque area in the atherosclerotic carotid plaque.

Morphological and functional changes occur in the DCs during maturation and post maturation DCs become more potent APCs with increased expression of major histocompatibility complex II (MHC II) antigen and numerous long dendrites. Fascin, an actin bundling protein regulating the cytoskeletal rearrangement and cytoskeleton-cell membrane interaction, plays an important role in the formation of the dendrites and these dendrites makes connections in between to make a crosslink structure. Dendrite formation results in the expression of fascin in the DCs [31, 33]. Phagocytosis of the pathogen by immature DCs results in DC maturation. The matured DCs (fascin+) migrate to lymphoid tissue and induce differentiation of naïve T lymphocytes to effector T cells. Th1 cells and cytotoxic cells mediate secretion of pro-inflammatory cytokines and cell destruction during pro-atherogenic process, while Th2 cells mediate both the pro- and anti-atherogenic effect [6]. We also found an increased number of pDCs in symptomatic carotid plaques, which is in agreement with previous reports [8, 34–36] and the positivity of these DCs for fascin shows the crucial role of mature DCs in plaque destabilization.

We observed increased co-localization of HLA-DR+ and fascin + cells with TREM-1 in symptomatic compared to asymptomatic carotid plaques. There are increasing evidence for the role of hypoxia in the pathogenesis of atherosclerosis and links between inflammatory and hypoxia signaling and development of atherosclerosis. Hypoxia is also present in macrophage-rich regions of carotid atherosclerosis plaque in human and helps in the advancement of the lesion [37–40]. Polymorphonuclear neutrophils (PMNs) are thought to play an important role in the development of atherosclerosis by increased endothelial adhesion or infiltration [41], increased production of reactive oxygen species [42] or via interaction with non-immune and immune cells namely monocytes, macrophage, DCs, platelets and smooth muscle cells [43]. TREM-1 is present on the surface of monocytes and PMNs and plays an important role in activation of these cells and production of reactive oxygen species. Increased expression of TREM-1 on PMN in chronic limb ischemia has been reported. TREM-1 plays an important role in interaction of monocytes and PMN with platelet [17, 18, 44, 45] and is upregulated in inflammation, severe infection and sepsis [46, 47]. These findings suggest that TREM-1 plays a critical role in atherogenesis and its expression on DCs might be related to plaque instability. In this study, we also found an increased immunoreactivity and mRNA expression of TREM-1 in S compared to AS and this increased reactivity may be due to the infiltrated monocytes, neutrophils and increased number of DCs in symptomatic atherosclerotic carotid plaque. Thus, the inhibition of TREM-1 could be a target to stabilize atherosclerotic plaque, as has been recently supported by studies in experimental atherosclerotic mice [48].

TREM-1 activates neutrophils and monocytes through transmembrane adapter protein, DAP-12, and induces the secretion of pro-inflammatory cytokines and chemokines [15]. Activation of monocytes through TREM-1 results in its differentiation to immature DCs and improved ability for T cell proliferation and production of INF-γ [49]. Langer et al. [16] reported that DCs recruitment in the atheroma is mediated by platelets and TREM-1 is expressed on platelet surface. TREM-1 involvement in neutrophil activation after neutrophil-platelet interaction results in respiratory burst activity and secretion of IL-8 [17–18], and monocytes activation after monocyte-platelet interaction results in secretion of pro-inflammatory cytokines [19]. Bosco et al. [13] reported that hypoxia can induce TREM-1 expression on mDCs and TREM-1 is a new biomarker of hypoxic mDCs involved in an inflammatory process. Presence of mDCs and hypoxia in atherosclerotic plaques might trigger induction and activation of TREM-1 and thus the pathogenesis, progression and vulnerability of carotid plaques. This could be strongly supported by our findings of significantly increased expression of TREM-1 in symptomatic carotid plaques and co-localization with HLA-DR+ and fascin+ cells.

Intima of the large vessels in human consists of smooth muscle cells and stellate-shaped cells. The α-SMA is a marker for vascular smooth muscles. These smooth muscle cells regulate the tonicity of the arterial wall. The α-SMA+ cells are involved in the production and maintenance of extracellular matrix, mainly elastin and collagen [30]. In this study, the symptomatic plaques also exhibited lower number of VSMCs, as we reported previously [6, 7]. IFN-γ, the inducer of MHC class II antigen in smooth muscle cells and macrophages is produced by T lymphocytes and the MHC II positivity of the neighboring cells indicate the important role of T lymphocytes in atherosclerotic process [9]. It has been demonstrated that IFN-α release in the plaque stimulates naïve CD4+ T cells to produce IFN-γ and to express TNF-related apoptosis-inducing ligand in an antigen-dependent manner, enabling them to kill VSMCs [50–51], which may help to explain the reduced number of VSMCs in S plaque and thus plaque instability [5, 6, 12].

We found significantly increased expression of TREM-2 and co-localization with α-SMA in asymptomatic while significantly increased TREM-1 expression but decreased α-SMA expression in symptomatic carotid plaque. These finding suggest that inflammation may play an important role in decreased expression of α-SMA and VSMCs depletion. It has also been reported that in significantly expanded atherosclerotic plaque lesion, exogenous administration of IFN-γ results in increased number of T cells and MHC II-positive cells, including macrophages and dendritic cells, and inhibition or ablation of IFN-γ results in reduced plaque cellularity and progression, and results in plaque stabilization involving decreased expression of cytokines, chemokines, adhesion molecules, MMPs and CD40 [16]. Role of TREM-1 in inflammatory diseases, such as COPD, inflammation in vascular bed, inflammatory bowel disease and gastric ulcers, is well established. Since there is an important role of monocytes and macrophages in atherosclerosis and plaque instability, TREM-1 activation in atherosclerosis can be related to stages and extent of atherosclerotic disease. Hence, presence of high level of TREM-1 in carotid atherosclerotic plaque may reflect the presence of an unstable plaque in symptomatic patients [52, 53]. TREM-2, a structurally related gene to TREM-1, is expressed on monocyte-derived DCs and promotes their *invivo* activation. TREM-2 is involved in DC maturation, survival and activation resulting in amplification of DC response to pathogens [49, 54, 55]. Our results of increased immunoreactivity of TREM-2 in asymptomatic atherosclerotic plaques are in accordance with the studies showing increased number of matured DCs in atherosclerotic plaque. TREM-2 plays a role in the regulation of immunity and it expression on newly differentiated and activated macrophages that have been recruited to the peripheral circulation prevents further activation of macrophages [56].

Fixation destroys or shields most cell surface epitopes. This means that nearly all DC markers cannot be studied in formaldehyde-fixed human specimens [57]. So, to circumvent this limitation, we have defined the plaque DC subsets and enumerated them by multi-colored flow cytometry. Interestingly, both mDCs and pDCs were found in carotid atherosclerotic plaques. However, in AS carotid plaques, the majority of the DCs had a phenotype of mDC_1_ and mDC_2_ with a few pDCs. However, in the symptomatic carotid plaques the majority of the DCs had a phenotype of pDCs with very few mDC_1_ or mDC_2_. Previous studies have shown that circulating mDC_1_ and pDC numbers are diminished in coronary artery disease and there is increased intimal DC count with evolving plaque stages, possibly due to increased recruitment from circulation to plaque areas [58].

Myeloid DCs and pDCs appear to be distinct in phenotype and function [4, 57]. The mDC_1_ and mDC_2_ descend from the myeloid lineage and express blood DC antigen BDCA-1 (CD1c) and BDCA-3 (CD141), respectively. The mDC_1_ produce and secrete mainly IL-12 in response to bacterial component while mDC_2_ produce IL-12 and type-1 IFN-β and also has a superior capacity to produce T_H_1 response when compared to mDC_1_ [10, 59, 60]. Unlike the mDCs, the pDCs express BDCA-2 (CD303) and CD123 and are specialized in innate antiviral immune response by producing large amount of type-1 INF-α and INF-β in response to toll like receptor (TLR)-7 and TLR9 stimulation [61]. Apart from responding to different pathogen-associated molecular patterns and secreting different cytokines, mDCs and pDCs also differ in their migratory pattern. It is assumed that mDCs are the conventional DCs that infiltrate peripheral tissue while pDCs migrate directly from the blood into lymphoid organ [4]. Our results are consistent with other studies that have shown localized pDC markers in carotid artery plaques, primarily in the shoulder region, where they cluster with myeloid DCs [50, 62, 63]. In atherosclerotic tissue, IFN-α transcripts correlate with plaque instability and TNF-α expression [50, 63], which may explain higher number of pDCs observed in symptomatic plaque.

Furthermore, pDCs may act as inflammatory amplifiers, as plaque DCs respond to IFN-α by producing markedly higher amount of pro-inflammatory mediators, such as cytokines and MMPs [63]. In addition to viral components, nucleotides released from necrotic and apoptotic cells in plaque may bind and stimulate TLR7 or TLR9, leading to the induction of IFN-α production by pDCs in the presence of antimicrobial peptides released from inflammatory cells [64]. Conversely, pDCs can also act as tolerogenic cells by expressing inducible tolerogenic enzyme indoleamine 2, 3-dioxygenase, the inducible co-stimulator ligand, and /or the programmed death-1 ligand, which mediate regulatory T cell development [61, 65, 66]. Nonetheless, the exact role of pDCs in atherosclerosis remains to be conclusively determined.

## Conclusion

In this study, we found that the majority of the DCs in S plaque belong to pDC phenotype. The increased presence of pDCs suggests an enhanced IFN-α secretion resulting in increased TNF-related apoptosis-inducing ligand-mediated apoptosis of coronary VSMCs and consequent exacerbation of vascular inflammation leading to plaque instability. The precise role of pDCs is debatable due to complexity of pDCs functions in atherosclerosis. It will be interesting to determine how increased levels of potential antigens from the necrotic core and the alteration in the T-cell response affect pDC function and recruitment during atherogenesis or *vice-versa*. Additional studies are warranted to determine the pro-inflammatory versus tolerogenic functions of pDCs in regard to degree of hyperlipidemia and the lesion progression in atherosclerotic plaques. We also demonstrated the over-expression of TREM-1 and co-localization of over-expressed TREM-1 with HLA-DR+ and fascin+ cells in S compared to AS plaques, indicating a possible role of TREM-1 in plaque instability along with DCs. These results support the theory that TREM-1 inhibition may be a novel strategy for enhancing plaque stability in carotid artery atherosclerosis. However, further studies are warranted to examine the expression of TREM-1 and TREM-2, and the effect of pro- and anti-inflammatory cytokines and growth factors on the expression of TREMs in isolated individual DCs subsets.

## Disclaimer

The content of this article is solely the responsibility of the authors and does not necessarily represent the official views of the NIH.

## References

[1] Alsheikh-Ali AA, Kitsios GD, Balk EM, Lau J, Ip S. The vulnerable atherosclerotic plaque: scope of the literature. Ann Intern Med. 2010;153(6):387–95. 10.7326/0003-4819-153-6-201009210-00272 20713770

[2] Hansson GK. Inflammation, atherosclerosis, and coronary artery disease. N Engl J Med. 2005;352(16):1685–95. 15843671 10.1056/NEJMra043430

[3] Thorp E, Subramanian M, Tabas I. The role of macrophages and dendritic cells in the clearance of apoptotic cells in advanced atherosclerosis. Eur J Immunol. 2011;41(9):2515–8. 10.1002/eji.201141719 21952808 PMC3289088

[4] Van Vre EA, Van Brussel I, Bosmans JM, Vrints CJ, Bult H. Dendritic cells in human atherosclerosis: from circulation to atherosclerotic plaques. Mediators Inflamm. 2011;2011:941396 10.1155/2011/941396 21976788 PMC3184502

[5] Andersson J, Libby P, Hansson GK. Adaptive immunity and atherosclerosis. Clin Immunol. 2010;134(1):33–46. 10.1016/j.clim.2009.07.002 19635683

[6] Dhume AS, Soundararajan K, Hunter WJ 3rd, Agrawal DK. Comparison of vascular smooth muscle cell apoptosis and fibrous cap morphology in symptomatic and asymptomatic carotid artery disease. Ann Vasc Surg. 2003;17(1):1–8. 12522697 10.1007/s10016-001-0331-1

[7] Dhume AS and Agrawal DK: Inability of vascular smooth muscle cells to proceed beyond S phase of cell cycle and increased apoptosis in symptomatic carotid artery disease. J Vasc Surg. 2003; 38(1): 155–61. 12844105 10.1016/s0741-5214(02)75463-3

[8] Dietel B, Cicha I, Voskens CJ, Verhoeven E, Achenbach S, Garlichs CD. Decreased numbers of regulatory T cells are associated with human atherosclerotic lesion vulnerability and inversely correlate with infiltrated mature dendritic cells. Atherosclerosis. 2013;230(1):92–9 10.1016/j.atherosclerosis.2013.06.014 23958259

[9] Libby P. Inflammation in atherosclerosis. Arterioscler Thromb Vasc Biol. 2012;32(9):2045–51. 10.1161/ATVBAHA.108.179705 22895665 PMC3422754

[10] Shortman K, Liu Y-J. Mouse and human dendritic cell subtypes. Nat Rev Immunol. 2002;2(3):151–61. 11913066 10.1038/nri746

[11] Shortman K, Naik SH. Steady-state and inflammatory dendritic-cell development. Nat Rev Immunol. 2007;7(1):19–30. 17170756 10.1038/nri1996

[12] Moran EP, Agrawal DK. Increased expression of inhibitor of apoptosis proteins in atherosclerotic plaques of symptomatic patients with carotid stenosis. Exp Mol Pathol. 2007;83(1):11–6. 17208224 10.1016/j.yexmp.2006.09.006 PMC2745193

[13] Bosco MC, Pierobon D, Blengio F, Raggi F, Vanni C, Gattorno M, et al Hypoxia modulates the gene expression profile of immunoregulatory receptors in human mature dendritic cells: identification of TREM-1 as a novel hypoxic marker in vitro and in vivo. Blood. 2011;117(9): 2625–39 10.1182/blood-2010-06-292136 21148811

[14] Zanzinger K, Schellack C, Nausch N, Cerwenka A. Regulation of triggering receptor expressed on myeloid cells 1 expression on mouse inflammatory monocytes. Immunology. 2009;128(2):185–95. 10.1111/j.1365-2567.2009.03091.x 19740375 PMC2767308

[15] Pelham CJ, Agrawal DK: Emerging roles for triggering receptor expressed on myeloid cells (TREM) receptor family signaling in inflammatory diseases. Expert Rev Clin Immunol. 10(2): 243–256, 2014 10.1586/1744666X.2014.866519 24325404

[16] Langer HF, Daub K, Braun G, Schonberger T, May AE, Schaller M, et al Platelets recruit human dendritic cells via Mac-1/JAM-C interaction and modulate dendritic cell function in vitro. Arterioscler Thromb Vasc Biol. 2007;27(6):1463–70. 17379836 10.1161/ATVBAHA.107.141515

[17] May AE, Seizer P, Gawaz M. Platelets: inflammatory firebugs of vascular walls. Arterioscler Thromb Vasc Biol. 2008;28(3):s5–10. 10.1161/ATVBAHA.107.158915 18174454

[18] Haselmayer P, Grosse-Hovest L, von Landenberg P, Schild H, Radsak MP. TREM-1 ligand expression on platelets enhances neutrophil activation. Blood. 2007;110(3):1029–35. 17452516 10.1182/blood-2007-01-069195

[19] De Meyer I, Martinet W, De Meyer GR. Therapeutic strategies to deplete macrophages in atherosclerotic plaques. Br J Clin Pharmacol. 2012;74(2):246–63. 10.1111/j.1365-2125.2012.04211.x 22309283 PMC3630745

[20] Berk BC, Weintraub WS, Alexander RW. Elevation of C-reactive protein in "active" coronary artery disease. Am J Cardiol. 1990;65(3):168–72. 2296885 10.1016/0002-9149(90)90079-g

[21] Schindhelm RK, van der Zwan LP, Teerlink T, Scheffer PG. Myeloperoxidase: a useful biomarker for cardiovascular disease risk stratification? Clin Chem. 2009;55(8):1462–70. 10.1373/clinchem.2009.126029 19556446

[22] Brea D, Sobrino T, Blanco M, Fraga M, Agulla J, Rodriguez-Yanez M, et al Usefulness of haptoglobin and serum amyloid A proteins as biomarkers for atherothrombotic ischemic stroke diagnosis confirmation. Atherosclerosis. 2009;205(2):561–7. 10.1016/j.atherosclerosis.2008.12.028 19171342

[23] Ishida Y, Migita K, Izumi Y, Nakao K, Ida H, Kawakami A, et al The role of IL-18 in the modulation of matrix metalloproteinases and migration of human natural killer (NK) cells. FEBS Lett. 2004; 569(1–3):156–60. 15225625 10.1016/j.febslet.2004.05.039

[24] Hermus L, Lefrandt JD, Tio RA, Breek JC, Zeebregts CJ. Carotid plaque formation and serum biomarkers. Atherosclerosis. 2010;213(1):21–9. 10.1016/j.atherosclerosis.2010.05.013 20627248

[25] Malaud E, Merle D, Piquer D, Molina L, Salvetat N, Rubrecht L, et al Local carotid atherosclerotic plaque proteins for the identification of circulating biomarkers in coronary patients. Atherosclerosis. 2014;233(2):551–8. 10.1016/j.atherosclerosis.2013.12.019 24530963

[26] Cipollone F, Fazia M, Mincione G, Iezzi A, Pini B, Cuccurullo C, et al Increased expression of transforming growth factor-beta1 as a stabilizing factor in human atherosclerotic plaques. Stroke. 2004;35(10):2253–7. 15297631 10.1161/01.STR.0000140739.45472.9c

[27] Rao VH, Kansal V, Stoupa S, Agrawal DK: MMP-1 and MMP-9 Regulate Epidermal Growth Factor-dependent Collagen Loss in Human Carotid Plaque Smooth Muscle Cells. *Physiol Reports (Heart & Circ Physiol)* 2014; 2(2): e00224. 10.1002/phy2.224 PMC3966234 24744893

[28] Rao VH, Rai V, Stoupa S, Agrawal DK: Blockade of Ets-1 attenuates epidermal growth factor-dependent collagen loss in human carotid plaque smooth muscle cells. Am J Phyiol.–Heart & Circ Physiol 2015 9 15; 309(6):H1075–86. 10.1152/ajpheart.00378.2015 PMC4591361 26254334

[29] Abebe W, Cavallari N, Agrawal DK, Rowley J, Thorpe PE, Hunyer WJ, Edwards JD. Functional and morphological assessment of rat aorta stored in University of Wisconsin and Eurocollins solutions. Transplantation. 1993;56(4):808–16. 8212198 10.1097/00007890-199310000-00006

[30] Bobryshev YV, Moisenovich MM, Pustovalova OL, Agapov II, Orekhov AN. Widespread distribution of HLA-DR-expressing cells in macroscopically undiseased intima of the human aorta: a possible role in surveillance and maintenance of vascular homeostasis. Immunobiology. 2012;217(5):558–68. 10.1016/j.imbio.2011.03.014 21601938

[31] Yamashiro S. Functions of fascin in dendritic cells. Crit Rev Immunol. 2012;32(1):11–21. 22428853 10.1615/critrevimmunol.v32.i1.20

[32] Mallat Z, Ait-Oufella H, Tedgui A. Regulatory T-cell immunity in atherosclerosis. Trends Cardiovasc Med. 2007;17(4):113–8. 17482092 10.1016/j.tcm.2007.03.001

[33] Al-Alwan MM, Rowden G, Lee TD, West KA. Fascin is involved in the antigen presentation activity of mature dendritic cells. J Immunol. 2001;166(1):338–45. 11123310 10.4049/jimmunol.166.1.338

[34] Yilmaz A, Lochno M, Traeg F, Cicha I, Reiss C, Stumpf C, et al Emergence of dendritic cells in rupture-prone regions of vulnerable carotid plaques. Atherosclerosis. 2004;176(1):101–10. 15306181 10.1016/j.atherosclerosis.2004.04.027

[35] Bobryshev YV, Lord RS. Mapping of vascular dendritic cells in atherosclerotic arteries suggests their involvement in local immune-inflammatory reactions. Cardiovasc Res. 1998;37(3):799–810. 9659465 10.1016/s0008-6363(97)00229-0

[36] Bobryshev YV, Lord RS, Parsson H. Immunophenotypic analysis of the aortic aneurysm wall suggests that vascular dendritic cells are involved in immune responses. Cardiovasc Surg. 1998; 6(3):240–9. 9705095 10.1177/096721099800600305

[37] Marsch E, Sluimer JC, Daemen MJ. Hypoxia in atherosclerosis and inflammation. Curr Opin Lipidol. 2013;24(5):393–400. 10.1097/MOL.0b013e32836484a4 23942270

[38] Hulten LM, Levin M. The role of hypoxia in atherosclerosis. Curr Opin Lipidol. 2009;20(5):409–14. 10.1097/MOL.0b013e3283307be8 19644366

[39] Song D, Fang G, Mao SZ, Ye X, Liu G, Gong Y, et al Chronic intermittent hypoxia induces atherosclerosis by NF-kappaB-dependent mechanisms. Biochim Biophys Acta. 2012; 1822(11):1650–9. 10.1016/j.bbadis.2012.07.010 22846605

[40] Douglas RM, Bowden K, Pattison J, Peterson AB, Juliano J, Dalton ND, et al Intermittent hypoxia and hypercapnia induce pulmonary artery atherosclerosis and ventricular dysfunction in low density lipoprotein receptor deficient mice. J Appl Physiol (1985). 2013;115(11):1694–704. 23990245 10.1152/japplphysiol.00442.2013 PMC3882740

[41] Weber C, Zernecke A, Libby P. The multifaceted contributions of leukocyte subsets to atherosclerosis: lessons from mouse models. Nat Rev Immunol. 2008;8(10):802–15. 10.1038/nri2415 18825131

[42] Mazor R, Shurtz-Swirski R, Farah R, Kristal B, Shapiro G, Dorlechter F, et al Primed polymorphonuclear leukocytes constitute a possible link between inflammation and oxidative stress in hyperlipidemic patients. Atherosclerosis. 2008;197(2):937–43. 17869258 10.1016/j.atherosclerosis.2007.08.014

[43] van Gils JM, Zwaginga JJ, Hordijk PL. Molecular and functional interactions among monocytes, platelets, and endothelial cells and their relevance for cardiovascular diseases. J Leukoc Biol. 2009;85(2):195–204. 10.1189/jlb.0708400 18948548

[44] Klesney-Tait J, Turnbull IR, Colonna M. The TREM receptor family and signal integration. Nat Immunol. 2006;7(12):1266–73. 17110943 10.1038/ni1411

[45] Ford JW, McVicar DW. TREM and TREM-like receptors in inflammation and disease. Curr Opin Immunol. 2009;21(1):38–46. 10.1016/j.coi.2009.01.009 19230638 PMC2723941

[46] Bouchon A, Facchetti F, Weigand MA, Colonna M. TREM-1 amplifies inflammation and is a crucial mediator of septic shock. Nature. 2001;410(6832):1103–7. 11323674 10.1038/35074114

[47] Gibot S, Kolopp-Sarda MN, Bene MC, Bollaert PE, Lozniewski A, Mory F, et al A soluble form of the triggering receptor expressed on myeloid cells-1 modulates the inflammatory response in murine sepsis. J Exp Med. 2004;200(11):1419–26. 15557347 10.1084/jem.20040708 PMC2211948

[48] Joffre J, Potteaux S, Boufenzer A, Loyer X, Mellak S, Laurans L, et al Inhibition and Genetic Deficiency of Trem-1 Receptor Reduces Development of Experimental Atherosclerosis in Mice. Circulation. 2014;130(Suppl 2):A18838–A.

[49] Bleharski JR, Kiessler V, Buonsanti C, Sieling PA, Stenger S, Colonna M, et al A role for triggering receptor expressed on myeloid cells-1 in host defense during the early-induced and adaptive phases of the immune response. J Immunol. 2003;170(7):3812–8. 12646648 10.4049/jimmunol.170.7.3812

[50] Niessner A, Sato K, Chaikof EL, Colmegna I, Goronzy JJ, Weyand CM. Pathogen-sensing plasmacytoid dendritic cells stimulate cytotoxic T-cell function in the atherosclerotic plaque through interferon-alpha. Circulation. 2006;114(23):2482–9. 17116765 10.1161/CIRCULATIONAHA.106.642801

[51] Manthey HD, Zernecke A. Dendritic cells in atherosclerosis: functions in immune regulation and beyond. Thromb Haemost. 2011;106(5):772–8. 10.1160/TH11-05-0296 21901235

[52] Hermus L, Schuitemaker JH, Tio RA, Breek JC, Slart RH, de Boef E, et al Novel serum biomarkers in carotid artery stenosis: useful to identify the vulnerable plaque? Clin Biochem. 2011;44(16):1292–8. 10.1016/j.clinbiochem.2011.08.1141 21939648

[53] Kim J, Gozal D, Bhattacharjee R, Kheirandish-Gozal L. TREM-1 and pentraxin-3 plasma levels and their association with obstructive sleep apnea, obesity, and endothelial function in children. Sleep. 2013;36(6):923–31. 10.5665/sleep.2726 23729936 PMC3649834

[54] Colonna M, Facchetti F. TREM-1 (triggering receptor expressed on myeloid cells): a new player in acute inflammatory responses. J Infect Dis. 2003;187 Suppl 2:S397–401. 12792857 10.1086/374754

[55] Bouchon A, Hernandez-Munain C, Cella M, Colonna M. A DAP12-mediated pathway regulates expression of CC chemokine receptor 7 and maturation of human dendritic cells. J Exp Med. 2001;194(8):1111–22. 11602640 10.1084/jem.194.8.1111 PMC2193511

[56] Turnbull IR, Gilfillan S, Cella M, Aoshi T, Miller M, Piccio L, et al Cutting edge: TREM-2 attenuates macrophage activation. J Immunol. 2006;177(6):3520–4. 16951310 10.4049/jimmunol.177.6.3520

[57] Van Vre EA, Bosmans JM, Van Brussel I, Maris M, De Meyer GR, Van Schil PE, et al Immunohistochemical characterisation of dendritic cells in human atherosclerotic lesions: possible pitfalls. Pathology. 2011;43(3):239–47. 10.1097/PAT.0b013e328344e266 21436634

[58] Van Vre EA, Hoymans VY, Bult H, Lenjou M, Van Bockstaele DR, Vrints CJ, et al Decreased number of circulating plasmacytoid dendritic cells in patients with atherosclerotic coronary artery disease. Coron Artery Dis. 2006;17(3):243–8. 16728874 10.1097/00019501-200605000-00007

[59] Jarrossay D, Napolitani G, Colonna M, Sallusto F, Lanzavecchia A. Specialization and complementarity in microbial molecule recognition by human myeloid and plasmacytoid dendritic cells. Eur J Immunol. 2001;31(11):3388–93. 11745357 10.1002/1521-4141(200111)31:11<3388::aid-immu3388>3.0.co;2-q

[60] Kadowaki N, Ho S, Antonenko S, Malefyt RW, Kastelein RA, Bazan F, et al Subsets of human dendritic cell precursors express different toll-like receptors and respond to different microbial antigens. J Exp Med. 2001;194(6):863–9. 11561001 10.1084/jem.194.6.863 PMC2195968

[61] Colonna M, Trinchieri G, Liu YJ. Plasmacytoid dendritic cells in immunity. Nat Immunol. 2004; 5(12):1219–26. 15549123 10.1038/ni1141

[62] Sorrentino R, Morello S, Pinto A. Plasmacytoid dendritic cells: from heart to vessels. Int J Vasc Med. 2010;2010:430318 10.1155/2010/430318 21152192 PMC2989744

[63] Niessner A, Shin MS, Pryshchep O, Goronzy JJ, Chaikof EL, Weyand CM. Synergistic proinflammatory effects of the antiviral cytokine interferon-alpha and Toll-like receptor 4 ligands in the atherosclerotic plaque. Circulation. 2007;116(18):2043–52. 17938289 10.1161/CIRCULATIONAHA.107.697789

[64] Marshak-Rothstein A, Busconi L, Rifkin IR, Viglianti GA. The stimulation of Toll-like receptors by nuclear antigens: a link between apoptosis and autoimmunity. Rheum Dis Clin North Am. 2004; 30(3):559–74, ix. 15261341 10.1016/j.rdc.2004.04.005

[65] Matta BM, Castellaneta A, Thomson AW. Tolerogenic plasmacytoid DC. Eur J Immunol. 2010; 40(10):2667–76. 10.1002/eji.201040839 20821731 PMC3974856

[66] Ochando JC, Homma C, Yang Y, Hidalgo A, Garin A, Tacke F, et al Alloantigen-presenting plasmacytoid dendritic cells mediate tolerance to vascularized grafts. Nat Immunol. 2006; 7(6):652–62. 16633346 10.1038/ni1333

